# Supplementary material for: The mitoepigenome responds to stress, suggesting novel mito-nuclear interactions in vertebrates
Source: BMC Genomics. 2023 Sep 22;24:561. doi: 10.1186/s12864-023-09668-9 (PMC10515078; doi:10.1186/s12864-023-09668-9)
Supplement: Supplementary file 1 — Additional file 1: Fig. S1. Locations of DNA-binding motifs for HNF4 (a) and ZNF324 (b) (taken from a human database and locations identified using FIMO) within the mitochondrial genomes of Gallus gallus, Homo sapiens, Alligator mississippiensis, Danio rerio and Drosophila melanogaster. Fig. S2. Schematic of the bioinformatics pipeline used to identify the differentially methylated based in a CpN context throughout the mitochondrial genome. Table S1. Table of the raw sequencing read data, corresponding to the whole genome (WG), the mitochondrial chromosome (MT) and representing true mitochondrial genome reads after the removal of nuclear mitochondrial pseudogenes (numts). Table S2. Start base, stop base, length, gene location within the mtDNA, mean direction of cytosine methylation and sequence of the mtDMRs identified in our analysis across different pairwise statistical comparisons. Contrasts representing statistical comparisons are as follows; male and female control birds (MC vs. FC), male and female stressed birds (MS vs. FS), male control vs. male stress birds (MC vs. MS) and female control vs. female stress birds (FC vs. FS). Table S3. Base composition within mtDMRs across our pairwise statistical comparisons compared to the base composition of the mitochondrial genome (A = 30.3%, T = 23.8%, C = 32.5%, G = 13.5%). Contrasts representing statistical comparisons are as follows; male and female control birds (MC vs. FC), male and female stressed birds (MS vs. FS), male control vs. male stress birds (MC vs. MS) and female control vs. female stress birds (FC vs. FS). Table S4. A comparison of the occurrences CpN dinucleotides within mtDMRs. CpA, CpT, CpC and CpC occurrences within mtDMRs of the mitcochondrial DNA of male and female chickens exposed to circadian light stress. Values are expressed in relation to pairwise statistical comparisons between Male control vs. female control (MC vs. FC), male stress vs. female stress (MS vs. FS), male control vs. male stres [file 12864_2023_9668_MOESM1_ESM.zip › JL_2023_supplementary_materials.docx]

Supplementary Materials for

**The mitoepigenome responds to stress, suggesting novel mito-nuclear interactions in vertebrates**

John Lees *et al.*

*Corresponding author. Email: [carlos.guerrero.bosagna@ebc.uu.se](mailto:carlos.guerrero.bosagna@ebc.uu.se)


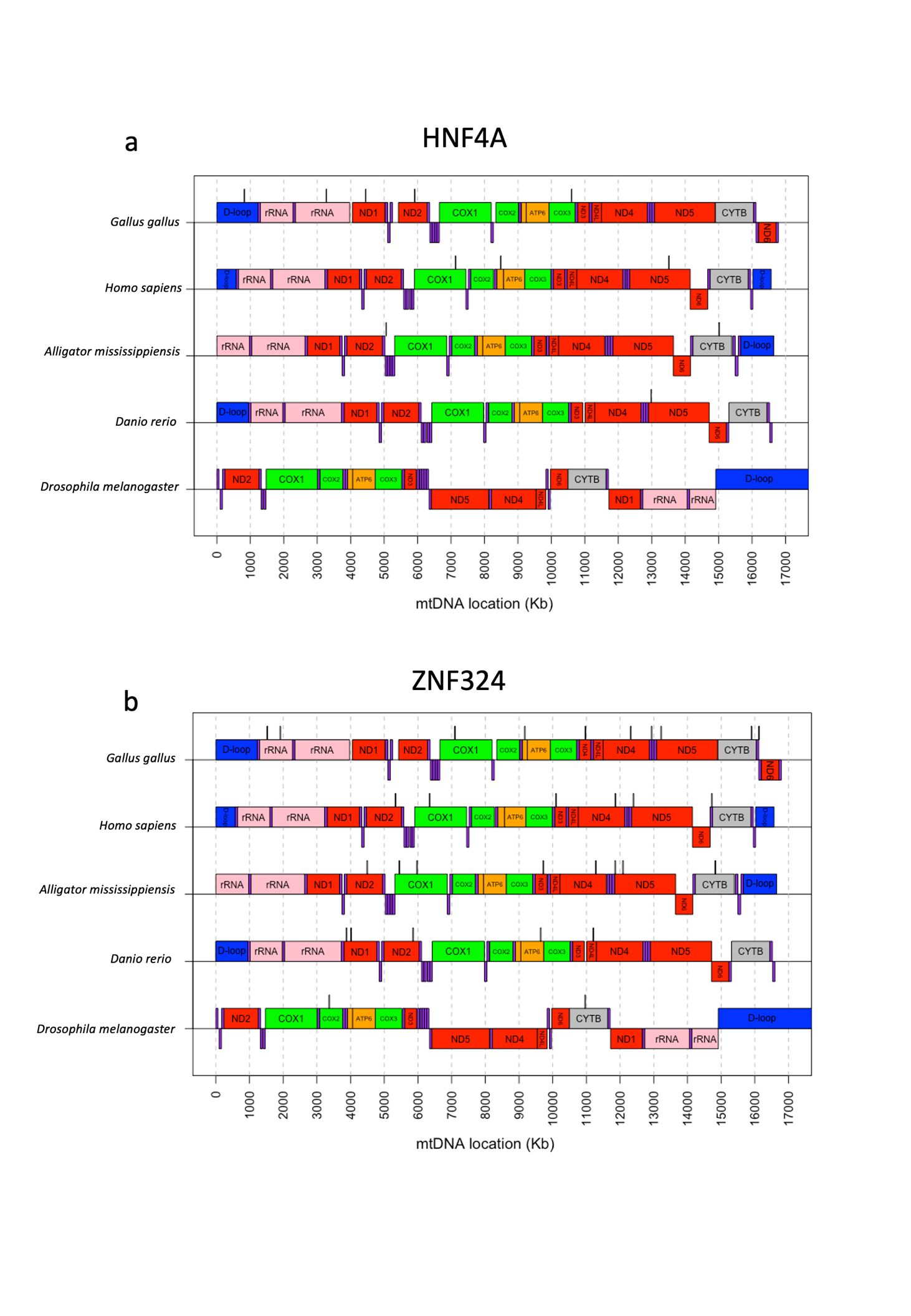


Fig. S1.

Locations of DNA-binding motifs for HNF4 (a) and ZNF324 (b) (taken from a human database and locations identified using FIMO) within the mitochondrial genomes of *Gallus gallus, Homo sapiens, Alligator mississippiensis, Danio rerio* and *Drosophila* *melanogaster*.


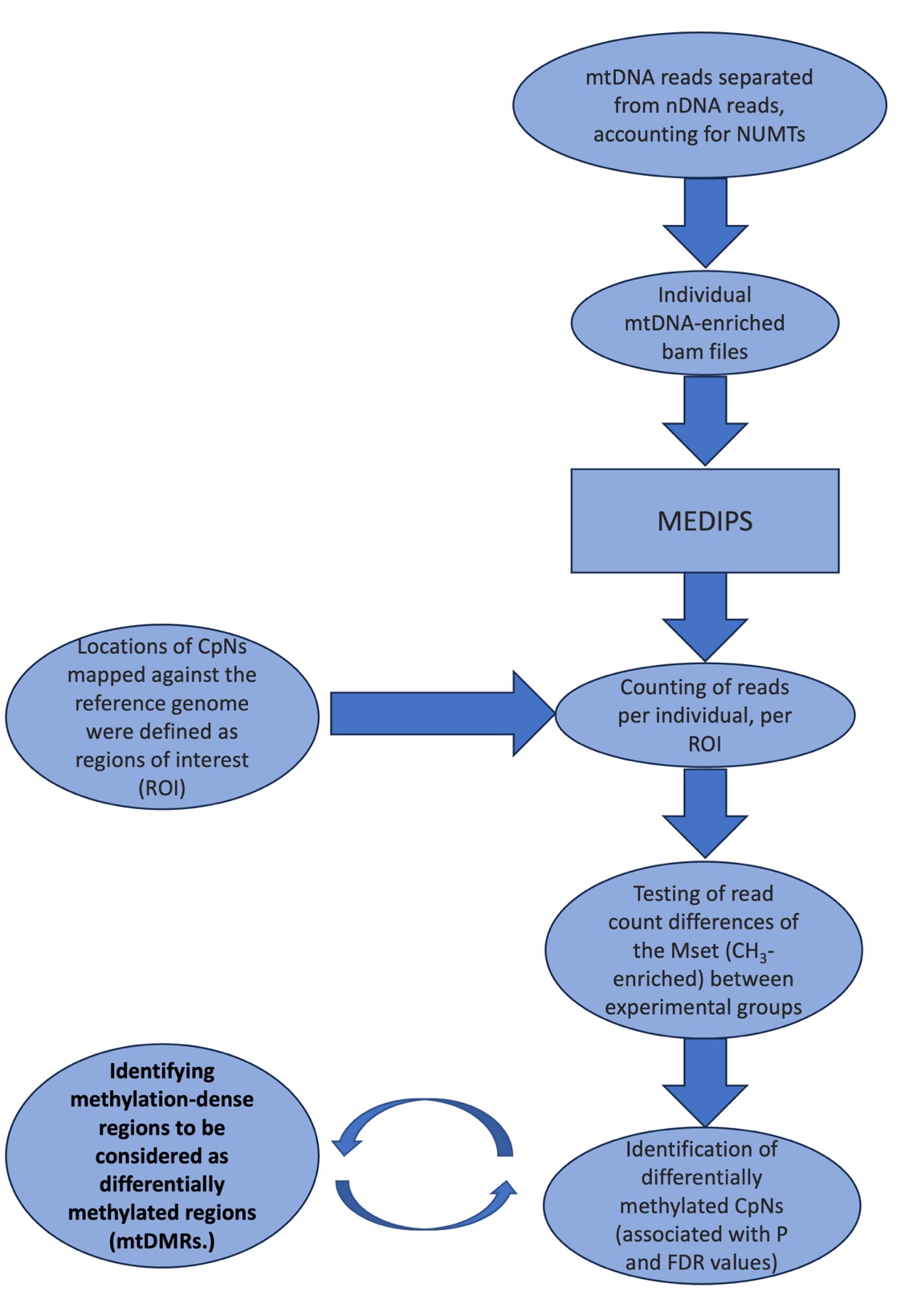


Fig. S2. Schematic of the bioinformatics pipeline used to identify the differentially methylated based in a CpN context throughout the mitochondrial genome.

Table S1.(separate file)

Table of the raw sequencing read data, corresponding to the whole genome (WG), the mitochondrial chromosome (MT) and representing true mitochondrial genome reads after the removal of nuclear mitochondrial pseudogenes (numts).

**Table S2.**

Start base, stop base, length, gene location within the mtDNA, mean direction of cytosine methylation and sequence of the mtDMRs identified in our analysis across different pairwise statistical comparisons. Contrasts representing statistical comparisons are as follows; male and female control birds (MC vs. FC), male and female stressed birds (MS vs. FS), male control vs. male stress birds (MC vs. MS) and female control vs. female stress birds (FC vs. FS).

**Table S3.**

Base composition within mtDMRs across our pairwise statistical comparisons compared to the base composition of the mitochondrial genome (A = 30.3%, T = 23.8%, C = 32.5%, G = 13.5%). Contrasts representing statistical comparisons are as follows; male and female control birds (MC vs. FC), male and female stressed birds (MS vs. FS), male control vs. male stress birds (MC vs. MS) and female control vs. female stress birds (FC vs. FS).

**Table S4.**

A comparison of the occurrences CpN dinucleotides within mtDMRs. CpA, CpT, CpC and CpC occurrences within mtDMRs of the mitcochondrial DNA of male and female chickens exposed to circadian light stress. Values are expressed in relation to pairwise statistical comparisons between Male control vs. female control (MC vs. FC), male stress vs. female stress (MS vs. FS), male control vs. male stress (MC vs. MS) and female control vs. female stress (FC vs. FS). Chi squared p-values > 0.05 indicate no difference between the number of CpNs with differential sequencing coverage in comparison to the total number of CpNs present within the mtDMRs.

**Table S5.**

Frequency matrix for human ATF4 used to determine ATF4 locations within diverse taxa. Source: http://floresta.eead.csic.es/footprintdb/index.php?motif=591111e9f6326e96b78d40f0820b6e8d

**Table S6.**

Frequency matrix for human ZNF324 used to determine ZNF324 locations within diverse taxa. Source: https://jaspar2022.genereg.net/matrix/MA1977.1/

**Table S7.**

Frequency matrix for human HNF4A used to determine HNF4A locations within diverse taxa. Source: https://jaspar2022.genereg.net/matrix/MA0114.4/

**Tabel S8.**

Locations of DNA-binding motifs for ATF4 (taken from a human database and locations identified using FIMO) within the mitochondrial genomes of *Gallus gallus, Homo sapiens, Alligator mississippiensis, Danio rerio* and *Drosophila melanogaster*.

**Tabel S9.**

Locations of DNA-binding motifs for ZNF324 (taken from a human database and locations identified using FIMO) within the mitochondrial genomes of *Gallus gallus, Homo sapiens, Alligator mississippiensis, Danio rerio* and *Drosophila melanogaster.*

**Tabel S10.**

Locations of DNA-binding motifs for HNF4 (taken from a human database and locations identified using FIMO) within the mitochondrial genomes of *Gallus gallus, Homo sapiens, Alligator mississippiensis, Danio rerio* and *Drosophila melanogaster*.

Table S11.

Table used to calculate the CpG enrichment score of samples based upon MeDIP samples in comparison to the reference genome.

Data S1. (separate file)

The differentially methylated CpN dinucleotides identified through pairwise statistical comparisons and their locations within the mitochondrial genome. Contrasts representing statistical comparisons are as follows; male and female control birds (MC vs. FC), male and female stressed birds (MS vs. FS), male control vs. male stress birds (MC vs. MS) and female control vs. female stress birds (FC vs. FS).
