# Supplementary material for: The mitoepigenome responds to stress, suggesting novel mito-nuclear interactions in vertebrates
Source: BMC Genomics. 2023 Sep 22;24:561. doi: 10.1186/s12864-023-09668-9 (PMC10515078; doi:10.1186/s12864-023-09668-9)
Supplement: Supplementary file 1 — Additional file 1: Fig. S1. Locations of DNA-binding motifs for HNF4 (a) and ZNF324 (b) (taken from a human database and locations identified using FIMO) within the mitochondrial genomes of Gallus gallus, Homo sapiens, Alligator mississippiensis, Danio rerio and Drosophila melanogaster. Fig. S2. Schematic of the bioinformatics pipeline used to identify the differentially methylated based in a CpN context throughout the mitochondrial genome. Table S1. Table of the raw sequencing read data, corresponding to the whole genome (WG), the mitochondrial chromosome (MT) and representing true mitochondrial genome reads after the removal of nuclear mitochondrial pseudogenes (numts). Table S2. Start base, stop base, length, gene location within the mtDNA, mean direction of cytosine methylation and sequence of the mtDMRs identified in our analysis across different pairwise statistical comparisons. Contrasts representing statistical comparisons are as follows; male and female control birds (MC vs. FC), male and female stressed birds (MS vs. FS), male control vs. male stress birds (MC vs. MS) and female control vs. female stress birds (FC vs. FS). Table S3. Base composition within mtDMRs across our pairwise statistical comparisons compared to the base composition of the mitochondrial genome (A = 30.3%, T = 23.8%, C = 32.5%, G = 13.5%). Contrasts representing statistical comparisons are as follows; male and female control birds (MC vs. FC), male and female stressed birds (MS vs. FS), male control vs. male stress birds (MC vs. MS) and female control vs. female stress birds (FC vs. FS). Table S4. A comparison of the occurrences CpN dinucleotides within mtDMRs. CpA, CpT, CpC and CpC occurrences within mtDMRs of the mitcochondrial DNA of male and female chickens exposed to circadian light stress. Values are expressed in relation to pairwise statistical comparisons between Male control vs. female control (MC vs. FC), male stress vs. female stress (MS vs. FS), male control vs. male stres [file JL_2023_table S1.pdf › JL_2023_table S1.pdf]

Samples	MappedReads			%Mapped reads (MT-numt/MG)*100	Depth		
	WG	MT	MT - numt		WG	MT	MT - numt
MS011	12 585 379.00	22 680.00	21 768.00	0.17	6.24474	162.294	155.522
MC035	16 337 942.00	31 755.00	28 831.00	0.18	7.41619	239.93	222.354
FC039	12 684 884.00	13 368.00	12 518.00	0.10	6.42543	102.727	97.1344
MC040	13 354 421.00	17 809.00	17 157.00	0.13	6.12635	125.978	121.134
FS042	16 622 205.00	23 068.00	21 760.00	0.13	6.63821	175.721	166.767
MS048	9 689 850.00	25 756.00	23 867.00	0.25	5.56829	192.126	180.416
MS065	12 429 416.00	21 828.00	20 954.00	0.17	6.31002	165.162	158.326
MC073	12 222 382.00	23 602.00	22 678.00	0.19	6.31621	176.374	169.024
FC105	14 440 432.00	21 867.00	20 498.00	0.14	6.60052	169.258	159.975
FS109	11 548 040.00	24 431.00	22 215.00	0.19	5.71807	183.432	170.807
FS119	14 659 737.00	34 246.00	30 871.00	0.21	6.41795	256.347	236.749
FC122	12 447 136.00	32 252.00	30 732.00	0.25	5.31088	244.535	233.241
**Average**	13 251 819	24 389	22 821	0.17	6.26	182.82	172.62
**SD**	1 977 806	6 016	5 346	0.05	0.55	45.94	41.97

Samples	bp Sequenced			% bp Sequenced(MT-numt/MG)*100	Breadth (bp Sequenced/depth)			% breadth (MT-numt/MG)*100
	WG	MT	MT - numt		WG	MT	MT - numt	
MS011	3 005 324 323	2 720 703.00	2 606 388	0.09	481 256 918	16 764.04	16 759	0.003
MC035	4 607 508 626	4 024 832.00	3 728 655	0.08	621 277 047	16 775.03	16 769	0.003
FC039	3 838 250 967	1 723 238.00	1 627 292	0.04	597 353 168	16 774.93	16 753	0.003
MC040	2 930 082 997	2 113 277.00	2 032 024	0.07	478 275 482	16 774.97	16 775	0.004
FS042	3 601 282 954	2 947 712.00	2 797 515	0.08	542 508 139	16 774.96	16 775	0.003
MS048	3 270 347 022	3 222 529.00	3 022 501	0.09	587 316 218	16 773.00	16 753	0.003
MS065	3 662 120 271	2 770 596.00	2 655 915	0.07	580 365 874	16 775.02	16 775	0.003
MC073	3 795 688 755	2 958 670.00	2 835 370	0.07	600 944 040	16 774.98	16 775	0.003
FC105	3 976 979 889	2 833 879.00	2 678 460	0.07	602 525 239	16 742.95	16 743	0.003
FS109	3 471 375 831	3 077 080.00	2 859 988	0.08	607 088 726	16 775.04	16 744	0.003
FS119	3 783 334 872	4 300 216.00	3 971 462	0.10	589 492 731	16 774.98	16 775	0.003
FC122	3 667 732 688	4 083 252.00	3 894 660	0.11	690 607 336	16 698.03	16 698	0.002
**Average**	3 634 169 100	3 064 665.33	2 892 519	0.08	581 584 243	16 764.83	16 758	0.00
**SD**	448 019 676	768 625.02	702 120	0.02	58 415 531	23.03	23	0.00

Samples	% of GGA6		
	WG	MT	MT - numt
MS011	42.4	99.93	99.90
MC035	54.7	100.00	99.96
FC039	52.6	100.00	99.87
MC040	42.1	100.00	100.00
FS042	47.7	100.00	100.00
MS048	51.7	99.99	99.87
MS065	51.1	100.00	100.00
MC073	52.9	100.00	100.00
FC105	53.0	99.81	99.81
FS109	53.4	100.00	99.82
FS119	51.9	100.00	100.00
FC122	60.8	99.54	99.54
**Average**	51.18	99.94	99.90
**SD**	5.14	0.14	0.13

Affect of numts on total MT reads		
OnlyMT %	numt %	Total
95.978836	4.021164	100
90.792001	9.2079987	100
93.641532	6.358468	100
96.33893	3.6610702	100
94.329808	5.6701925	100
92.665787	7.3342134	100
95.995968	4.0040315	100
96.085078	3.9149225	100
93.739425	6.2605753	100
90.929557	9.0704433	100
90.144834	9.8551656	100
95.287114	4.712886	100
93.83	6.17	100
2.25	2.25	0

Legend

Acronym	Description
WG	Whole Genome
MT	mitochondrial chromossome
numt	Nuclear mitochondrial pseudo genes
